# Detection by Enzyme-Linked Immunosorbent Assay of Antibodies to *West Nile virus* in Birds

**DOI:** 10.3201/eid0809.020152

**Published:** 2002-09

**Authors:** Gregory D. Ebel, Alan P. Dupuis, David Nicholas, Donna Young, Joseph Maffei, Laura D. Kramer

**Affiliations:** * Wadsworth Center, New York State Department of Health, Slingerlands, New York, USA

**Keywords:** West Nile virus, enzyme-linked immunosorbent assay, IgG antibodies, birds, avian species

## Abstract

We adapted an indirect immunoglobulin G enzyme-linked immunosorbent assay to facilitate studies of *West Nile virus* (WNV) and evaluated its application to taxonomically diverse avian species. Anti-WNV antibodies were detected in 23 bird species, including many exotic species, demonstrating its value in studies of WNV epizootiology.

*West Nile virus* (WNV) is transmitted in an enzootic cycle between *Culex* spp. mosquitoes and their avian hosts (1–4). Sentinel birds have long been used in arbovirus surveillance (5–9), and serologic surveys of wild and captive birds are valuable in determining whether an arbovirus is present in a particular locality (10). While plaque-reduction neutralization tests (PRNT) are the standard for arbovirus serologic testing, they are frequently unavailable in many laboratories for several reasons: They generally require high levels of biocontainment; they are time-, labor-, and cost-intensive; and they require specialized technical expertise. A rapid serologic diagnostic assay suitable for screening large numbers of specimens and posing minimal biohazard would facilitate large-scale avian-based serologic surveillance for WNV. Accordingly, we sought to determine whether an indirect enzyme-linked immunosorbent assay (ELISA) designed to detect seroreactivity against *St. Louis encephalitis virus* (SLEV) and *Western equine encephalomyelitis virus* (WEEV) (11) could be modified to detect anti-WNV antibodies in taxonomically diverse wild-caught and captive avian species.

To produce ELISA antigen, Vero cells were infected with WNV and processed into antigen as described (12), with New York–derived reference stocks of WNV (31000365; see Ebel et al. [13] for source and sequence information). Fifty microliters of antigen diluted 1:100 in fresh coating buffer (0.015M Na_2_CO_3_, 0.035M NaHCO_3_, pH 9.6) was applied to each well of Immulon 1 (Dynatek Laboratories Inc, Winooski, VT) ELISA plates. Negative antigen (uninfected Vero cell lysate produced as described above) was placed in every third column of the plate (i.e., columns 1, 4, 7, 10), and positive antigen was placed in the remaining columns. The plate was then placed in a humid chamber, and antigen was allowed to bind overnight at 4°C. In the morning, antigen-containing solution was discarded, the plate was washed three times with phosphate-buffered saline (PBS) with 0.05% Tween, 100 µL blocking buffer (PBS with 0.05% Tween and 2.0% Casein) was added, and the plates were placed in a humid chamber in a 37°C incubator for 1 h. Following incubation, blocking solution was discarded and test samples, diluted 1:100 in PBS with 0.05% Tween, and 0.5% bovine albumin (PBS-T-BA), were applied to one negative and two positive antigen-containing wells. Plates with test specimens were returned to a humid chamber and incubated at 37°C for 1 h. Following incubation, plates were removed, washed as above, and 50 µL of horseradish peroxidase–conjugated goat anti-wild bird immunoglobulin (Ig) G (Bethyl Laboratories, Inc., Montgomery, TX), diluted 1:1000 in PBS-T-BA, was applied to each well. After incubation and washing as above, plates were developed with 50 µL of tetramethylbenzidine (TMB)-peroxidase substrate (Kirkegaard & Perry Laboratories, Inc., Gaithersburg, MD) for 7 min. The reactions were stopped with 50 µL of 1:20 H_2_PO_4_, and the optical density (OD) of each well was read at 450 nm. Blank (no test sera), positive, and negative controls were included on each plate. To compute the positive/negative (P/N) value of each sample, we divided the mean OD of positive antigen-containing wells by the OD of the negative antigen-containing wells. Samples with a P/N value >2 were considered positive and were tested further by PRNT.

Optimum concentrations of antigens for the ELISA were determined by applying known positive and negative chicken samples to wells containing serial twofold dilutions of antigen. Optimal concentrations were defined as those yielding the highest mean positive OD_450_ for two wells per sample containing positive antigen, divided by the OD_450_ for a single well containing that same serum specimen, and negative antigen (P/N value) for known positive samples and P/N values closest to unity (one) for known negative samples. Generally, a 1:100 dilution of the crude antigens was optimal. Using a similar strategy, we then determined the optimal serum dilutions for pigeon and wild bird sera.

Samples with a P/N value >2 were considered positive. PRNT were conducted on these specimens according to standard protocols (description following) (14). Specimens were confirmed positive if their 90% neutralization titer against WNV was at least fourfold greater than against SLEV, a closely related flavivirus that may cross-react with WNV antigens in screening assays (15,16).

Specimens for testing were either donated from the collection at the Bronx Zoo or collected during an avian surveillance project conducted in New York City during 2001. Avian blood samples were collected as whole blood and stored at 4°C, centrifuged for 10 min at maximum speed in a microcentrifuge, and serum was separated. In some cases, samples were collected and heparinized, and plasma was separated and stored as described previously.

PRNT testing was conducted according to standard protocols (14). Briefly, where sample quantities permitted, test sera were serially diluted from 1:5 through 1:160 in BA-1 diluent (M199H, 0.05M Tris pH 7.6, 1% bovine serum albumin, 0.35g/L sodium bicarbonate, 100 units/mL penicillin, 100 µg/mL streptomycin, and 1 µg/mL fungizone) and 100 µL was incubated overnight at 4°C with 100 µL of virus containing approximately 200 PFU of WNV (strain 31000365) or SLEV virus strain no. 59268 (Parton). If insufficient sample was available, higher starting dilutions (usually 1:10) were used. In the morning, 100 µL of each serum-virus mixture was added onto confluent monolayers of Vero cells and allowed to adsorb at 37^o^C for 1 h. Following incubation, a nutrient-agar overlay was added, and the plates were returned to the incubator. PRNT testing for WNV used a single basal medium Eagle–based overlay containing neutral red, while SLEV testing required application of a double overlay, the first without and the second with neutral red, applied 3 days after the first. Plaques were counted on the 3rd (WNV) or 5th (SLEV) day after the test was initiated. The highest dilution of serum neutralizing 90% of the inoculum as determined by back-titration was considered the neutralizing titer.

All statistical analyses were done with Microsoft Excel (Microsoft Corp., Redmond, WA).

The predictive value of a positive test (PVP) and of a negative test (PVN) were determined by using the sera of birds caught in mist nets during a WNV serologic survey conducted during the summer of 2001 (manuscript in preparation). Of 3,581 specimens tested, 233 (7%) were ELISA positive. Of these positive specimens, 163 (70%) were also positive by PRNT, for a PVP of 70%. Five additional ELISA-positive specimens yielded indeterminate results: although neutralizing antibody was detected by PRNT, a fourfold difference between WNV and SLEV titers was not detectable. To determine the PVN of the ELISA, 110 ELISA-negative specimens were tested for neutralizing antibody by PRNT. All ELISA-negative specimens were also negative by PRNT, yielding a PVN of 100%.

To determine whether this protocol detects antibodies against WNV in a wide range of bird species, we used our ELISA to test known positive (PRNT-confirmed) serum specimens from 23 different avian species. The indirect ELISA protocol detected anti-WNV antibody in all 23 species, representing 12 avian orders. All PRNT-positive specimens contained ELISA antibody to WNV (Table). Species that were negative by ELISA were uniformly negative by PRNT. One domestic chicken that had been experimentally infected with SLEV had a positive P/N ELISA result and a positive PRNT result. The infection was confirmed as SLEV since the SLEV titer on this specimen was fourfold greater than the WNV titer (data not shown). P/N values were not correlated with either PRNT titer (coeff.=0.44) or with the natural log of the PRNT titer (coeff.=0.30) (data not shown).

**Table:** Comparison of serologic assay results with reactivity across a range of avian orders and species

Common Name	Species	Order	Indirect ELISA P/N^a^	PRNT titer
House Sparrow # 1	*Passer domesticus*	Passeriformes	3.0	40
House Sparrow # 2	*P. domesticus*	Passeriformes	3.0	20
House Sparrow # 3	*P. domesticus*	Passeriformes	7.2	80
Northern Mockingbird	*Mimus polyglottos*	Passeriformes	2.1	>10
European Starling	*Sturnus vulgaris*	Passeriformes	3.0	40
American Crow #1	*Corvus brachyrhynchos*	Passeriformes	2.8	80
American Crow #2	*C. brachyrhynchos*	Passeriformes	2.0	20
Rock Dove #1	*Columba livia*	Columbiformes	2.9	80
Rock Dove #2	*C. livia*	Columbiformes	2.5	320
Rock Dove #3	*C. livia*	Columbiformes	7.6	10
White-naped Crane	*Grus vipio*	Gruiformes	5.3	>640
Waldrapp Ibis #1	*Gerontica eremita*	Ciconiiformes	9.4	320
Waldrapp Ibis #2	*G. eremita*	Ciconiiformes	11.4	640
Waldrapp Ibis #3	*G. eremita*	Ciconiiformes	4.0	80
Black Crowned Night Heron	*Nycticorax nycticorax*	Ciconiiformes	5.2	160
Flamingo #1	*Phoenicopterus chilensis*	Phoenicopteriformes	3.7	160
Flamingo #2	*P. chilensis*	Phoenicopteriformes	4.2	>640
Malay Great Argus	*Argusianus argus*	Galliformes	7.3	40
Kenya Crested Guineafowl	*Guttera edouardi*	Galliformes	6.7	80
Wild Turkey	*Meleagris gallopavo*	Galliformes	6.0	320
Bulwer’s Pheasant	*Lophura bulweri*	Galliformes	3.5	>640
American White Pelican	*Pelecanus erythrorhynchos*	Pelecaniformes	5.7	>640
Guanay Cormorant	*Phalacrocorax bougainvillii*	Pelecaniformes	2.1	80
Brown Pelican	*Pelecanus occidentalis*	Pelecaniformes	3.8	160
Domestic Goose	*Anser* sp*.*	Anseriformes	3.6	40
Trumpeter Swan	*Cygnus buccinator*	Anseriformes	4.0	160
Barred Owl	*Strix varia*	Strigiformes	2.6	160
Ostrich	*Struthio camelus*	Struthioniformes	3.9	640
Magellanic Penguin	*Spheniscus magellanicus*	Sphenisciformes	4.3	>640
Black-Necked Crane	*Grus nigricollis*	Gruiformes	2.4	80
Laughing Gull	*Larus atricilla*	Charadriiformes	6.9	320
Domestic Duck	*Anas* sp.	Anseriformes	1.1	<10
Canada Goose	*Branta canadensis*	Anseriformes	1.2	<20
Domestic chicken	*Gallus gallus*	Galliformes	0.9	<10
SLEV-positive chicken	*G. gallus*	Galliformes	2.5	20
WEEV-positive chicken	*G. gallus*	Galliformes	1.3	<20

WNV-negative specimens shown below bold line ^a^ELISA, enzyme-linked immunosorbent assay; P/N, positive/negative ratio; PRNT, plaque-reduction neutralization tests; SLEV, St. Louis encephalitis virus; WEEV, Western equine encephalomyelitis virus.

## Conclusions

The PVP of this assay appears to be somewhat lower than that of another reported ELISA protocol (17) and some other flavivirus serologic assays, such as PRNT, but is higher than that reported for the assay from which it was derived (11). The PVP of our test might have been higher had we more stringently evaluated our ELISA-positive specimens: a number of specimens had P/N values >2 because one of the two positive antigen wells was highly reactive. None of these specimens were confirmed by PRNT. The high values in the reactive well may have occurred as a result of technical error (e.g., splashing). Alternatively, the ELISA may be more sensitive than neutralization and may detect anti–WNV antibodies that PRNT does not. We always performed a confirmatory test to resolve true from false positives; nonetheless, this ELISA dramatically reduced the number of confirmatory tests we conducted during WNV surveillance in 2000 and 2001. Use of the ELISA described here yielded substantial cost reduction and time savings compared with screening specimens by PRNT.

This ELISA detected anti–WNV antibody in a taxonomically diverse array of captive and wild birds. In 23 species from 12 avian orders, IgG antibodies were detectable by using commercially available anti-wild bird horseradish peroxidase–conjugated antibodies. The breadth of the reactivity of this conjugate was surprising, given that it was generated by using IgG isolated from the sera of four species representing only four avian orders: Passeriformes, Columbiformes, Galliformes, and Anseriformes (11). Although this protocol has been documented to react broadly in an ELISA to detect SLEV antibody in 13 species representing seven orders (11), known positive sera from three orders (Ciconiiformes, Gruiformes, and Charadriiformes) were not detected. We obtained positive results for each of these orders. The reasons for this discrepancy in our results are not clear but may be related to differences in the antibody titer of the specimens we tested or to general differences in the immune response to WNV compared with SLEV. Alternatively, some of the measures we took to optimize our test (e.g., the substitution of tetramethylbenzidine peroxidase substrate for 2,2´-azino-di-[3-ethylbenzthiazoline-6-sulfonate]) may have increased the assay’s sensitivity, allowing detection of fewer bound conjugated antibodies, as may occur with test sera derived from divergent avian species. The lack of correlation between P/N values with PRNT titers is not surprising given that the P/N value was obtained from a single serum dilution and does not represent an endpoint titer. Although this serologic method should be evaluated for each avian order tested, our results demonstrate that this testing protocol is appropriate for WNV serologic surveys of free-ranging and captive sentinel birds.

## References

[1] Komar N. West Nile Viral Encephalitis. Rev Sci Tech. 2000;19:166–76. 11189714 10.20506/rst.19.1.1201

[2] Rappole JH, Derrickson SR, Hubalek Z. Migratory birds and spread of West Nile virus in the Western Hemisphere. Emerg Infect Dis. 2000;6:319–28. 10905964 10.3201/eid0604.000401 PMC2640881

[3] Ahmed T, Hayes CG, Baqar S. Comparison of vector competence for West Nile virus of colonized populations of *Culex tritaeniorhynchus* from southern Asia and the Far East. Southeast Asian J Trop Med Public Health. 1979;10:498–504. 538499

[4] Baqar S, Hayes CG, Murphy JR, Watts DM. Vertical transmission of West Nile virus by *Culex* and *Aedes* species mosquitoes. Am J Trop Med Hyg. 1993;48:757–62. 8333569 10.4269/ajtmh.1993.48.757

[5] Day JF, Carlson DB. The importance of autumn rainfall and sentinel flock location to understanding the epidemiology of St. Louis encephalitis virus in Indian River County, Florida. J Am Mosq Control Assoc. 1985;1:305–9. 2852706

[6] Day JF, Winner R, Parsons RE, Zhang JT. Distribution of St. Louis encephalitis viral antibody in sentinel chickens maintained in Sarasota County, Florida: 1978–1988. J Med Entomol. 1991;28:19–23. 2033613 10.1093/jmedent/28.1.19

[7] Morris CD, Baker WG, Stark L, Burgess J, Lewis AL. Comparison of chickens and pheasants as sentinels for eastern equine encephalitis and St. Louis encephalitis viruses in Florida. J Am Mosq Control Assoc. 1994;10:545–8. 7707062

[8] Reisen WK, Hardy JL, Presser SB. Evaluation of domestic pigeons as sentinels for detecting arbovirus activity in southern California. Am J Trop Med Hyg. 1992;46:69–79. 1536386 10.4269/ajtmh.1992.46.69

[9] Reisen WK, Lin J, Presser SB, Enge B, Hardy J. Evaluation of new methods for sampling sentinel chickens for antibodies to WEE and SLE Viruses. Proc Calif Mosq Vector Control Assoc. 1993;61:33–6.

[10] McLean RG, Mullenix J, Kerschner J, Hamm J. The house sparrow (*Passer domesticus*) as a sentinel for St. Louis encephalitis virus. Am J Trop Med Hyg. 1983;32:1120–9. 6312819 10.4269/ajtmh.1983.32.1120

[11] Chiles RE, Reisen WK. A new enzyme immunoassay to detect antibodies to arboviruses in the blood of wild birds. J Vector Ecol. 1998;23:123–35. 9879069

[12] Frazier CL, Shope RE. Detection of antibodies to alphaviruses by enzyme-linked immunosorbent assay. J Clin Microbiol. 1979;10:583–5. 393723 10.1128/jcm.10.4.583-585.1979 PMC273219

[13] Ebel GD, Dupuis AP II, Ngo KA, Nicholas DC, Kauffman EB, Jones SA, Partial genetic characterization of West Nile virus strains, New York State, 2000. Emerg Infect Dis. 2001;7:650–3. 11585527 10.3201/eid0704.010408 PMC2631742

[14] Lindsey HS, Calisher CH, Matthews JH. Serum dilution neutralization test for California group virus identification and serology. J Clin Microbiol. 1976;4:503–10. 1002829 10.1128/jcm.4.6.503-510.1976 PMC274511

[15] Calisher CH, Karabatsos N, Dalrymple JM, Shope RE, Porterfield JS, Westaway EG, Antigenic relationships between flaviviruses as determined by cross-neutralization tests with polyclonal antisera. J Gen Virol. 1989;70:37–43. 10.1099/0022-1317-70-1-37 2543738

[16] Mandl CW, Guirakhoo F, Holzmann H, Heinz FX, Kunz C. Antigenic structure of the flavivirus envelope protein E at the molecular level, using tick-borne encephalitis virus as a model. J Virol. 1989;63:564–71. 2463377 10.1128/jvi.63.2.564-571.1989 PMC247724

[17] Hall RA, Broom AK, Hartnett AC, Howard MJ, MacKenzie JS. Immunodominant epitopes on the NS1 protein of MVE and KUN viruses serve as targets for a blocking ELISA to detect virus-specific antibodies in sentinel animal serum. J Virol Methods. 1995;51:201–10. 10.1016/0166-0934(94)00105-P 7738140

